# Donor-Derived Regulatory Dendritic Cell Infusion Maintains Donor-Reactive CD4^+^CTLA4^hi^ T Cells in Non-Human Primate Renal Allograft Recipients Treated with CD28 Co-Stimulation Blockade

**DOI:** 10.3389/fimmu.2018.00250

**Published:** 2018-02-19

**Authors:** Mohamed B. Ezzelarab, Lien Lu, William F. Shufesky, Adrian E. Morelli, Angus W. Thomson

**Affiliations:** ^1^Department of Surgery, Starzl Transplantation Institute, University of Pittsburgh School of Medicine, Pittsburgh, PA, United States; ^2^Department of Immunology, University of Pittsburgh School of Medicine, Pittsburgh, PA, United States

**Keywords:** regulatory T cells, dendritic cells, co-stimulation blockade, renal allografts, non-human primates

## Abstract

Donor-derived regulatory dendritic cell (DCreg) infusion before transplantation, significantly prolongs renal allograft survival in non-human primates. This is associated with enhanced expression of the immunoregulatory molecules cytotoxic T-lymphocyte-associated antigen (Ag) 4 (CTLA4) and programmed cell death protein 1 (PD1) by host donor-reactive T cells. In rodents and humans, CD28 co-stimulatory pathway blockade with the fusion protein CTLA4:Ig (CTLA4Ig) is associated with reduced differentiation and development of regulatory T cells (Treg). We hypothesized that upregulation of CTLA4 by donor-reactive CD4^+^ T cells in DCreg-infused recipients treated with CTLA4Ig, might be associated with higher incidences of donor-reactive CD4^+^ T cells with a Treg phenotype. In normal rhesus monkeys, allo-stimulated CD4^+^CTLA4^hi^, but not CD4^+^CTLA4^med/lo^ T cells exhibited a regulatory phenotype, irrespective of PD1 expression. CTLA4Ig significantly reduced the incidence of CD4^+^CTLA4^hi^, but not CD4^+^CTLA4^med/lo^ T cells following allo-stimulation, associated with a significant reduction in the CD4^+^CTLA4^hi^/CD4^+^CTLA4^med/lo^ T cell ratio. In CTLA4Ig-treated renal allograft recipient monkeys, there was a marked reduction in circulating donor-reactive CD4^+^CTLA4^hi^ T cells. In contrast, in CTLA4Ig-treated monkeys with DCreg infusion, no such reduction was observed. In parallel, the donor-reactive CD4^+^CTLA4^hi^/CD4^+^CTLA4^med/lo^ T cell ratio was reduced significantly in graft recipients without DCreg infusion, but increased in those given DCreg. These observations suggest that pre-transplant DCreg infusion promotes and maintains donor-reactive CD4^+^CTLA4^hi^ T cells with a regulatory phenotype after transplantation, even in the presence of CD28 co-stimulation blockade.

## Introduction

Despite major advances in clinical organ transplantation, an important limitation remains patients’ life-long dependency on immunosuppressive drugs with associated increased risk of morbidity and mortality (1). Based on compelling pre-clinical evidence, regulatory immune cell therapy offers considerable potential for the development of protocols that may promote clinical transplant tolerance (2–5). Dendritic cells (DC) are uniquely well-equipped integrators and regulators of innate and adaptive immunity. They can promote antigen (Ag)-specific tolerance (3, 6–8) as well as regulate memory T cell (Tmem) responses (9–11), a major barrier to the induction of transplantation tolerance (12–15).

Recently, testing of CD28 co-stimulation blockade (Co-SB) using belatacept [cytotoxic T lymphocyte Ag 4:Ig (CTLA4Ig)] and a calcineurin inhibitor-free regimen (steroids and mycophenolate mofetil) in kidney transplant recipients, has resulted in an increased incidence of acute cellular rejection within 1 year after transplantation, despite superior graft function (16, 17). While allo-reactive Tmem are known to be Co-SB-resistant (18, 19), there is recent evidence that CTLA4Ig may reduce donor-reactive regulatory T cells (Treg)/Tmem ratios after transplantation and prevent Treg-dependent transplantation tolerance (20, 21).

We have reported previously (22) using a pre-clinical non-human primate (NHP) model, that administration of donor-derived, maturation-resistant, regulatory dendritic cells (DCreg) 1 week before transplantation, can significantly prolong major histocompatibility complex (MHC)-mismatched renal allograft survival in CTLA4Ig-treated recipients. Although no increase in regulatory T cells (Treg) was detected after transplantation, we observed increased Treg to Tmem ratios in the graft recipients given DCreg infusion. This was associated with upregulation of the co-inhibitory molecule CTLA4 (CD152) and programmed cell death protein 1 (PD1) by host CD4^+^ and CD8^+^ T cells in response to donor but not third party stimulation, suggesting donor-specific regulation of T cell responses (23).

Cytotoxic T-lymphocyte-associated Ag 4 is a critical negative regulator of T cell responses (24). It is expressed constitutively in Treg but only upregulated in conventional T cells after activation. CTLA4 deficiency in mice results in a lethal lymphoproliferative disease (25, 26), hence it is essential for maintaining T cell tolerance to self-Ags (27). In rodent models of organ transplantation, CTLA4 blockade accelerates acute rejection (28). Also, importantly, donor-reactive CD4^+^CD25^+^ Treg expressing high levels of CTLA4 promote allograft survival (29), an effect that is dependent on exposure to donor Ag before transplantation. Further, there is recent evidence that CTLA4 plays a critical role in Treg suppressive function (30). Moreover, CTLA4 can promote T cell suppressive function, even in the absence of forkhead box p3 (Foxp3) expression (31).

We hypothesized that upregulation of CTLA4 by rhesus allo-reactive CD4^+^ T cells might be associated with increased incidences of Treg, and that pre-transplant infusion of donor-derived DCreg might promote and maintain donor-reactive Treg after renal transplantation, despite host treatment with CTLA4Ig. In this study, we examined the relationship between expression of CTLA4 and a Treg phenotype by allo-stimulated rhesus monkey CD4^+^ T cells. We also investigated the influence of CD28 Co-SB on Treg development both *in vitro* and in CTLA4Ig-treated kidney allograft recipient monkeys, with or without DCreg infusion.

## Materials and Methods

### Experimental Animals

Indian male juvenile rhesus macaques (*Macacca mulatta*; 5–7 kg), obtained from the NIAID-sponsored colony (Yemasse, S.C.) were maintained in the Non-Human Primate Research Facility of the Department of Laboratory Animal Resources at the University of Pittsburgh School of Medicine. All procedures were approved by the University of Pittsburgh Institutional Animal Care and Use Committee. Experiments were conducted according to the guidelines set forth in the National Institutes of Health Guide for the Care and Use of Laboratory Animals. Specific environment enrichment was provided.

### Donor Leukapheresis and DCreg Generation

Leukapheresis and generation of donor-derived DCreg from circulating monocytes were performed as described (22, 32). Briefly, prospective transplant donors underwent cytokine treatment comprising granulocyte-macrophage colony-stimulating factor (GM-CSF) for 4 days, followed by granulocyte (G)-CSF for 4 days. Leukapheresis was performed using a dedicated COBE^®^ Spectra Apheresis System (Lakewood, CO, USA). Leukapheresis products were processed and stored in liquid N_2_ until needed for DCreg generation. DCreg were generated from purified CD14^+^ cells in recombinant human (rhu) GM-CSF + rhu IL-4 over 7 days, with the addition of Vitamin D3 on days 1 and 5, and rhu IL-10 on day 5 as previously described (32).

### DCreg Infusion, Renal Transplantation, and Immunosuppression

Renal transplantation was performed as described (22). Briefly, bilateral nephrectomy of native kidneys was performed before graft insertion and recipient pairs—i.e., control (no DCreg infusion; *n* = 4) and experimental (DCreg infusion; *n* = 4) received MHC-mismatched kidney grafts from the same donor. In the experimental group (Table 1), DCreg (3.5–10 × 10^6^/kg) were infused intravenously, 7 days before transplantation. All recipients in the control and DCreg groups received CTLA4Ig (abatacept; Bristol-Myers Squibb; Princeton, NJ, USA; 12.5 mg/kg i.v.) on day −7 and day −4, to further minimize risk of host sensitization. Four recipients (two in each group) received short-term co-stimulation blockade (CTLA4Ig; 20 mg/kg on days −1, 0, 2, 4, 7, and 10), while two recipients (two in each group) received long-term Co-SB (CTLA4Ig; 20 mg/kg on days −1, 0, 3, 7, 10, 14, 21, and 28, then 10 mg/kg on days 35, 42, 49, and 56). Intramuscular rapamycin (LC laboratories, Woburn, MA, USA) was given daily, starting on day −2 for 6 months. Whole blood trough levels were measured twice weekly and maintained between 10 and 15 ng/ml for the first month, between 5 and 10 ng/ml for the subsequent 4 months, and between 1 and 5 ng/ml for the sixth month. Immunosuppressive therapy was withdrawn completely at 6 months (22).

**Table 1:** Immunosuppressive regimen, regulatory dendritic cells (DCreg) infusion and kidney graft survival.

Group	Recipient	Immunosuppression		DCreg infused (×10^6^/kg body weight)	Experiment end-point (days post-transplant)^b^
		CTLA4-Ig^a^	Rapamycin		
Control (no DCreg infusion; *n* = 4)	M49 M112	Short-term	Trough level of 10–15 ng/ml maintained for 1 month, then 5–10 ng/ml for 4 months, and 1–5 ng/ml for the sixth month	N/A	75 39
	M143 M145	Long-term			28 57
DCreg infusion (*n* = 4)^c^	M113 M45	Short-term		5.2 10	300 118
	M148 M147	Long-term		4.0 3.5	109 160

^a^Details of short-term and long-term administration of cytotoxic T-lymphocyte-associated antigen (Ag) 4:Ig (CTLA4Ig) are explained in Section “Materials and Methods.”

*^b^Data published originally in The American Journal of Transplantation (22)*.

*^c^Donor-derived DCreg infused on day −7*.

### Mixed Leukocyte Reactions (MLRs)

Peripheral blood mononuclear cells (PBMC) were isolated from normal rhesus monkeys for *in vitro* studies. Unlabeled or carboxyfluorescein succinimidyl ester (CFSE; Molecular Probes, Eugene, OR, USA)-labeled PBMC were used as responders and CD2^+^ T cell-depleted allogeneic irradiated PBMC as stimulators, at 1:1 ratio. In some MLRs, CTLA4Ig was added (1 µg or 100 µg/ml) at the start of the culture. PBMC were also isolated before and after transplantation [post-operative days (POD) 28–56, unless otherwise specified], and co-cultured with either donor or third party cells. Data were acquired using an LSR II flow cytometer (Becton Dickinson, Franklin Lakes, NJ, USA) and analyzed with FlowJo software (Tree Star, San Carlos, CA, USA).

### Phenotypic Analysis of Allo-Reactive T Cells

The following fluorochrome-labeled monoclonal antibodies were used as described (22, 33) for cell surface or intracellular staining of rhesus T cells: CD3 PerCP-Cy5.5, CD4 APC-H7, CD28 APC-H7, CD127 (IL-7Rα) PE, CD45RA PE-Cy7, CTLA4/CD152 APC, and CTLA4/CD152 VB450 (all from BD Biosciences, San Jose, CA, USA), CD8α AF700, CD25 AF700, and Foxp3 VB421 (all from Biolegend, San Diego, CA, USA), and PD1/CD279 PE (from eBioscience, San Diego, CA, USA). Following surface staining for CD3, CD4, CD8, CD25, CD28, CD127, and PD1, cells were fixed and permeabilized for 45 min at 4°C using Fixation/Permeabilization buffer (eBioscience™; ref 00-5123-43). After fixation/permeabilization, cells were stained for CTLA4 and Foxp3. No antibodies were added to the co-cultures.

### Immunofluorescence Staining of Kidney Allografts

Tissues were collected from one recipient with no DCreg infusion (control group) on the day of euthanasia and from one recipient with DCreg infusion (experimental group) on POD 28 by open biopsy of the kidney graft. Tissues were embedded in O.C.T. (Miles), snap-frozen, and stored at −80°C. Cryostat sections (8–10 µm) were mounted on slides pre-coated with Vectabond (Vector) then fixed in 96% ethanol and allowed to dry. Sections were blocked successively with 5% goat serum and an avidin/biotin blocking kit (Vector). Next, sections were incubated with anti-human CD4 Ab (Dako; Clone 4B12, 1:100, overnight, 10°C), followed by Alexa Fluor 555-goat anti-mouse IgG (Molecular Probes, 1:400, 1 h, RT). The slides were then blocked with mouse irrelevant IgG1 (BD Biosystems, 1:100, 1 h, RT) and incubated successively with biotin anti-human CTLA4 (CD152) (clone BNI3, BD Biosystems, 1:100, 1 h, RT), followed by Streptavidin Dylight 488 (Jackson Immunoresearch, 1:400, 1 h, RT). Cell nuclei were stained with DAPI (Molecular Probes).

### Statistical Analyses

The significances of differences between groups were determined using Kruskal–Wallis one-way analysis of variance or Mann–Whitney *U* test, as appropriate. Significance was defined as *p* < 0.05.

## Results

### CTLA4 and PD1 Expression by CD4^+^ T Cells in Renal Allografts 1 Month Post-Transplant

**Figure 1:**
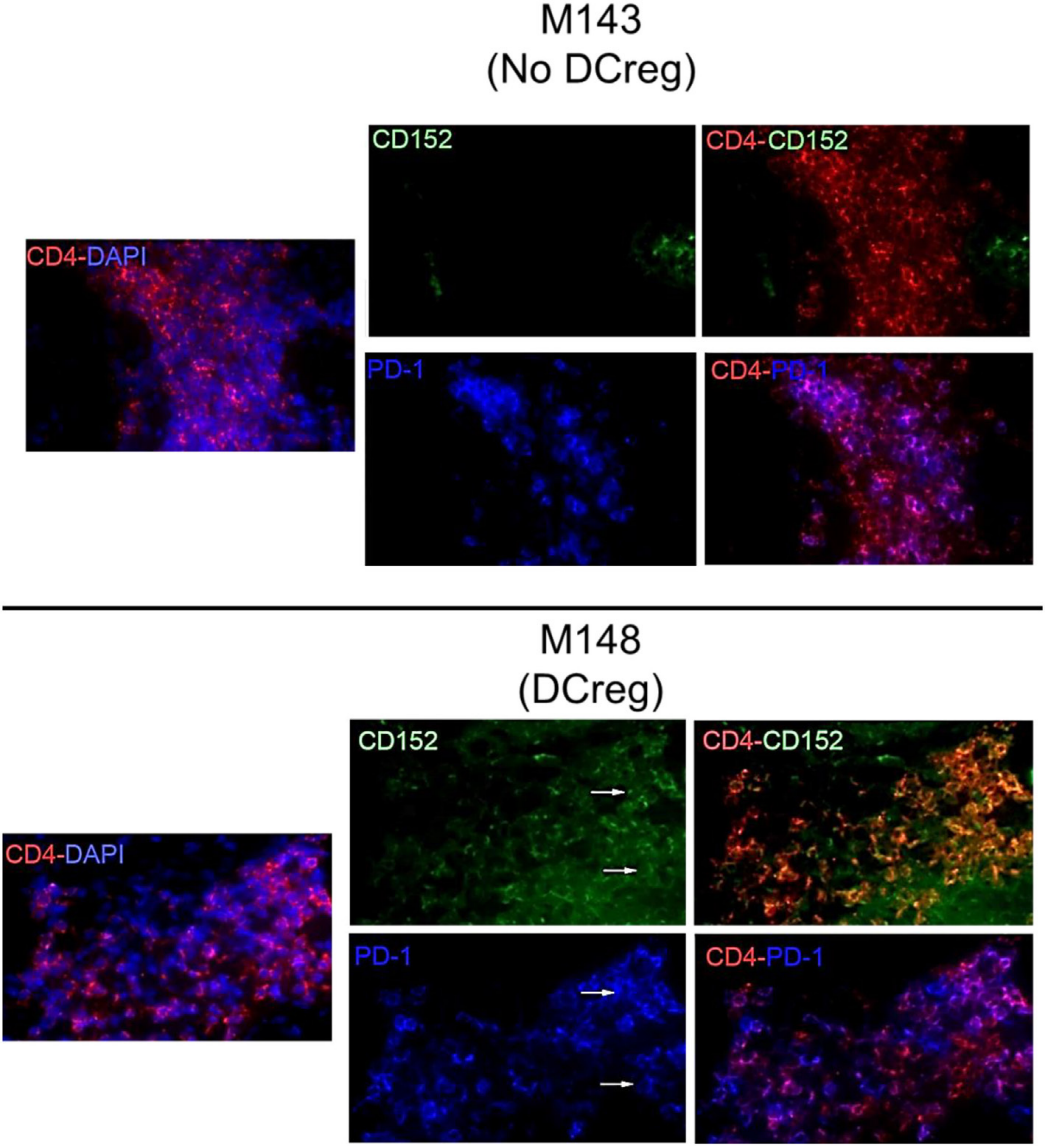
Cytotoxic T-lymphocyte-associated antigen (Ag) 4 (CTLA4) and programmed cell death protein 1 (PD1) expression by graft-infiltrating CD4^+^ T cells in rhesus renal allografts 1 month after transplantation. CTLA4 (CD152) and PD1 (CD279) expression was examined by immunofluorescence staining. Graft tissue from one monkey (M143) with no regulatory dendritic cells (DCreg) infusion at the time of graft rejection (post-operative day 28; POD 28) is shown. Tissue from a graft recipient given DCreg infusion (M148) was obtained by open kidney graft biopsy, also on POD 28. Co-localization of CTLA4 (green) or PD1 (blue) with CD4^+^ T cells (red) is shown (white arrows). Nuclei were stained with DAPI (blue). Slides were examined with a Nikon Eclipse E800 microscope equipped with a CCD camera (Nikon).

We have shown previously (22) that Tmem in rhesus renal allograft recipients given DCreg before transplant upregulate CTLA4 and PD1 expression in response to *ex vivo* donor but not third party stimulation. Additionally, graft-infiltrating CD8^+^ T cells were characterized by higher expression of CTLA4 and PD1 (33). Here, we hypothesized that graft-infiltrating CD4^+^ T cells in monkeys given DCreg infusion would also express high levels of CTLA4 and PD1. Thus, we examined the expression of CTLA4 and PD1 by graft-infiltrating CD4^+^ T cells 28 days post-transplant in monkeys given no DCreg infusion or DCreg infusion (Figure 1). With no DCreg infusion, graft-infiltrating CD4^+^ T cells showed minimal CTLA4 and PD1 expression. In contrast, strong expression of CTLA4 and PD1 by graft-infiltrating CD4^+^ T cells was observed in the recipient given DCreg infusion before transplantation. This observation is consistent with the upregulation of both CTLA4 and PD1 by circulating T cells from DCreg-infused graft recipients following *ex vivo* donor stimulation 28 days post-transplant (22).

### CTLA4 and PD1 Upregulation Correlates with Increased Regulatory CD4^+^ T Cell Marker Expression Following Allo-Stimulation *In Vitro*

**Figure 2:**
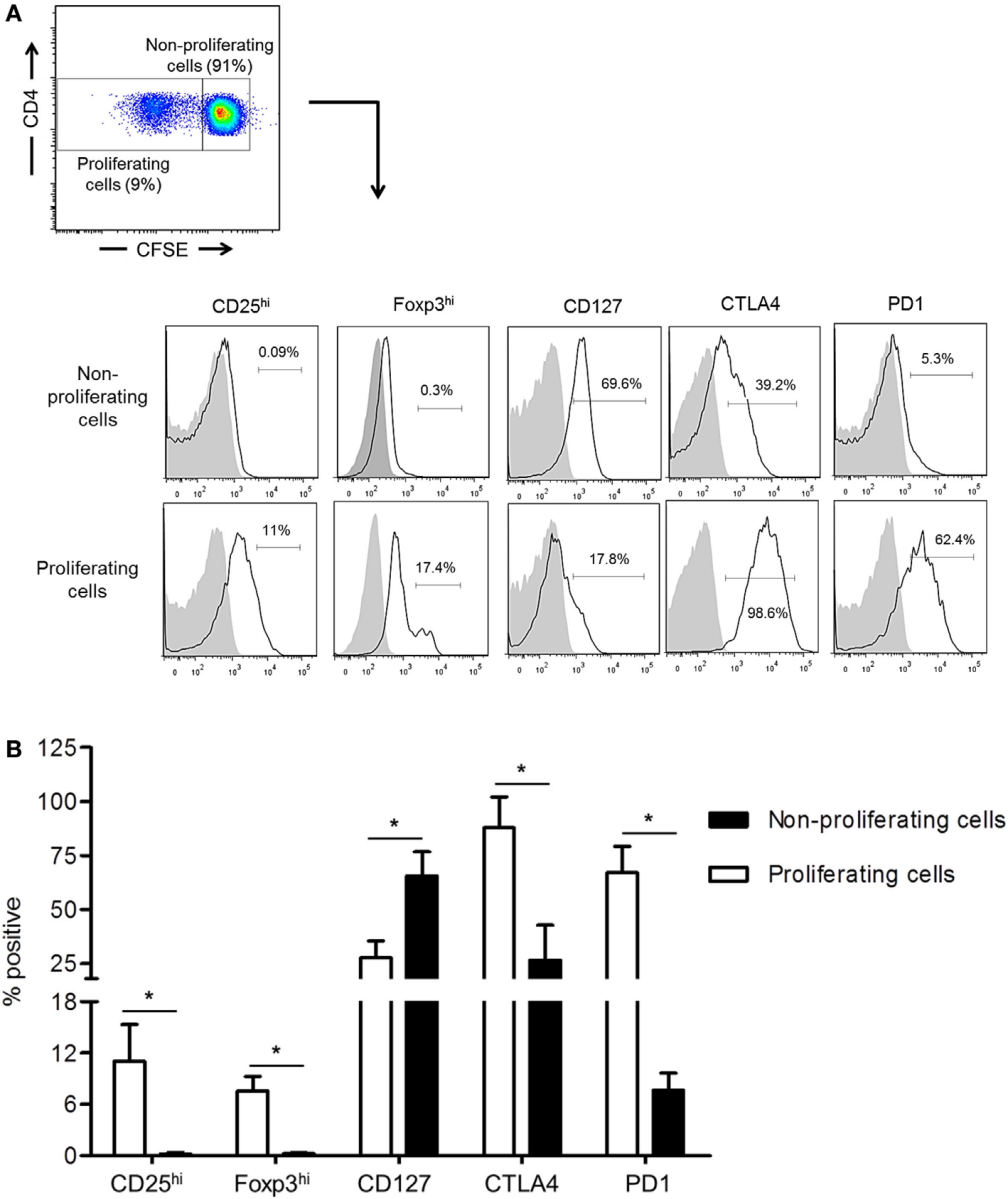
Upregulation of cytotoxic T-lymphocyte-associated antigen (Ag) 4 (CTLA4) and PD1 expression correlates with augmented regulatory T cells (Treg) phenotype by allo-stimulated rhesus CD4^+^ T cells. **(A)** The percentages of proliferating and non-proliferating CD4^+^ T cells expressing the Treg phenotype, CD25^hi^, Foxp3^hi^, and CD127^lo^, as well as CTLA4 and PD1 were evaluated in carboxyfluorescein succinimidyl ester (CFSE)-mixed leukocyte reaction. Responder peripheral blood mononuclear cells (PBMC) were co-cultured with allogeneic, T cell-depleted PBMC for 5 days. **(B)** Combined data from four individual monkeys are shown. Values are means + 1SD. *p < 0.05; only significant values are shown.

While the inhibitory molecules CTLA4 and PD1 are considered markers of T cell activation, exhaustion, and regulation (34, 35), they are also associated with the induction of Treg (36, 37). We examined their expression by normal rhesus CD4^+^ T cells together with Treg phenotype analysis based on CD25, CD127, and Foxp3 expression following their allo-stimulation for 5 days in CFSE-MLR. In comparison with non-proliferating cells, proliferating allo-reactive CD4^+^ T cells significantly upregulated CTLA4, PD1, CD25, and Foxp3 expression (*p* < 0.05), but significantly downregulated CD127 expression (*p* < 0.05) (Figure 2). These observations indicate that upregulation of CTLA4 and PD1 by allo-reactive CD4^+^ T cells is associated with an increased Treg phenotype.

### High CTLA4, but Not PD1 Expression Is Associated with a Regulatory CD4^+^ T Cell Phenotype in Normal Rhesus Monkeys

**Figure 3:**
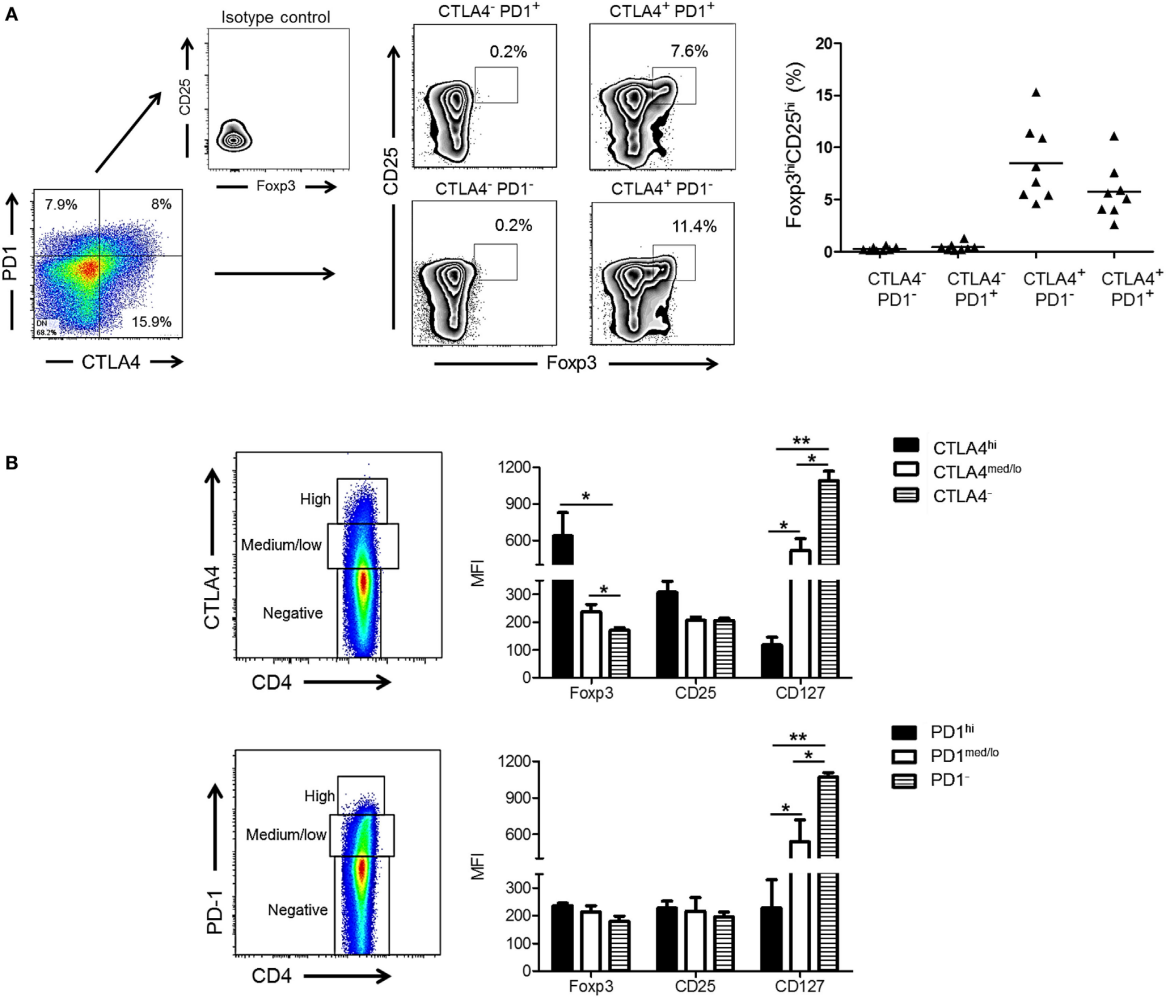
High cytotoxic T-lymphocyte-associated antigen (Ag) 4 (CTLA4), not programmed cell death protein 1 (PD1) expression correlates with a regulatory T cells (Treg) phenotype of allo-stimulated rhesus CD4^+^ T cells. **(A)** High CTLA4, but not PD1 expression correlates with CD4^+^ Treg in normal rhesus monkeys (left). Irrespective of PD1 expression, CTLA4^+^PD1^+^ and CTLA4^+^PD1^−^ CD4^+^ T cells exhibit a high incidence of dual CD25^hi^Foxp3^hi^ expression. Combined data from eight different monkeys are shown in the graph (right). **(B)** Treg were characterized based on expression of forkhead box p3 (Foxp3), CD25, and CD127 and evaluated in CD4^+^CTLA4^hi^, CD4^+^CTLA4^med/lo^, CD4^+^PD1^hi^, CD4^+^PD1^med/lo^ T cell populations after allo-stimulation in normal rhesus monkeys. Graphs show means + 1 SD from three independent experiments; *p < 0.05; **p < 0.01; only significant values are shown.

Cytotoxic T-lymphocyte-associated Ag 4 expression is critical for optimal Treg function and their ability to suppress T cell responses to allo-Ag (38, 39). In addition, Foxp3 is known to upregulate CTLA4 expression (40). We investigated whether expression of CTLA4 and/or PD1 by rhesus CD4^+^ T cells correlated with a higher frequency of Treg after allo-stimulation. As shown in Figure 3A, the incidence of CD25^hi^Foxp3^hi^ CD4^+^ T cells was significantly higher in the CTLA4^+^PD1^−^ and CTLA4^+^PD1^+^ populations than in the CTLA4^−^PD1^+^ and CTLA4^−^PD1^−^ populations. Thus, irrespective of PD1 expression, CTLA4 expression was associated with a higher incidence of CD25^hi^Foxp3^hi^ Treg.

Next, we evaluated the expression of Treg markers (Foxp3, CD25, and CD127) in relation to CTLA4 and PD1 expression by total (proliferating and non-proliferating) CD4^+^ T cells (Figure 3B). CD4^+^CTLA4^hi^ cells exhibited significantly higher levels of Foxp3 and CD25 than CD4^+^CTLA4^med/lo^ and CD4^+^CTLA4^neg^ T cells. Conversely, CD127 was expressed at the lowest level by CD4^+^CTLA4^hi^ T cells compared with CD4^+^CTLA4^med/lo^ and CD4^+^CTLA4^−^ T cells. In contrast, CD4^+^PD1^hi^ cells did not express higher levels of Foxp3 or CD25 than PD1^med/lo^ or PD1^neg^ CD4^+^ T cells. On the other hand, CD4^+^ PD1^−^ T cells expressed higher levels of CD127 than PD1^hi^ and PD1^med/lo^ CD4^+^ T cells. These data indicate that only high CTLA4 expression is associated with a Treg phenotype in normal rhesus monkeys.

### CD28 Co-SB Reduces CTLA4 Expression More Markedly Than PD1 Expression after Allo-Stimulation *In Vitro*

**Figure 4:**
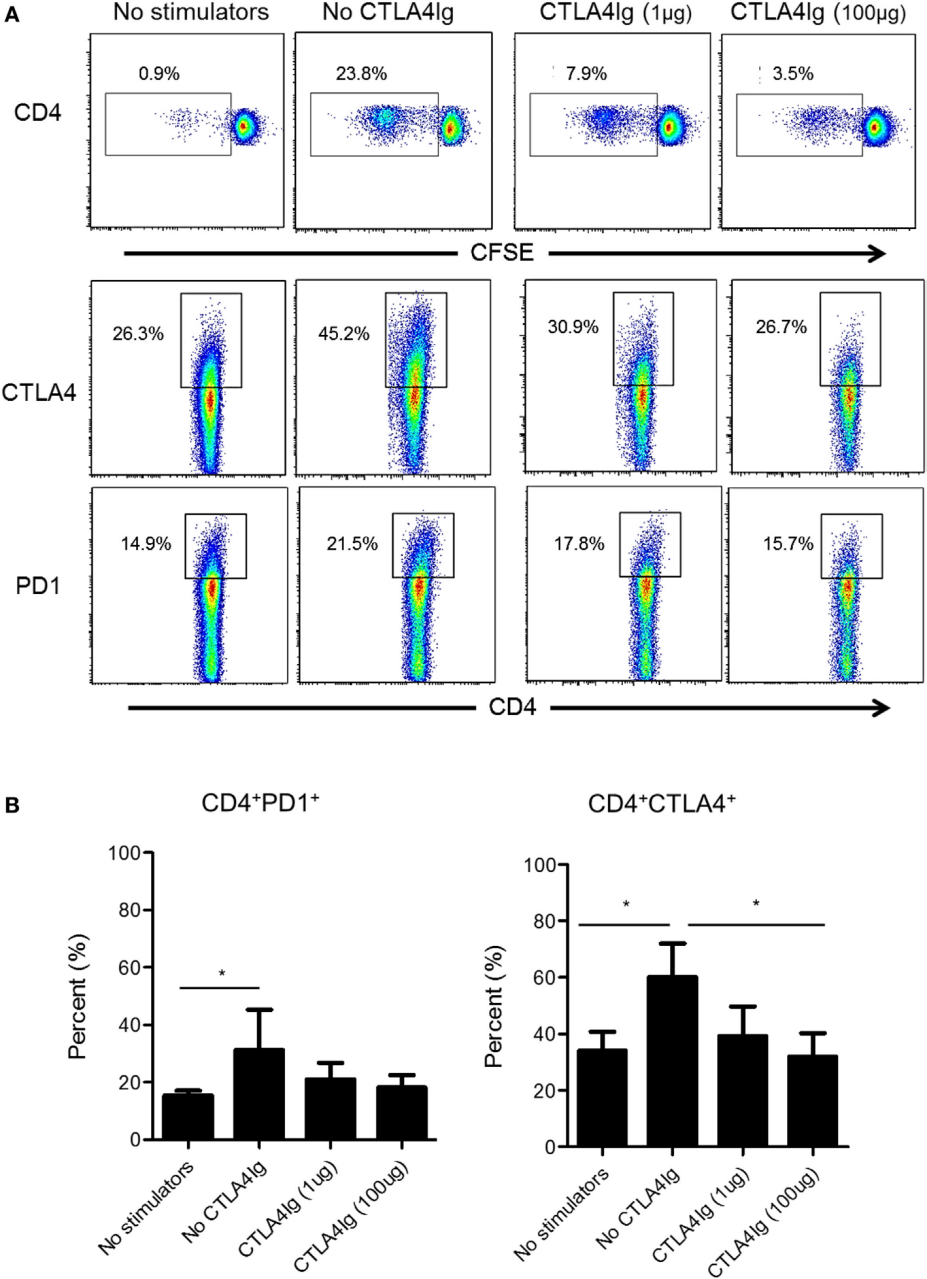
Cytotoxic T-lymphocyte-associated antigen (Ag) 4:Ig (CTLA4Ig) significantly reduces the incidence of allo-reactive CD4^+^CTLA4^hi^ T cells *in vitro*. **(A)** The percentages of CTLA4^+^ and programmed cell death protein 1 (PD1^+^) total CD4^+^ T cells were determined following allo-stimulation in mixed leukocyte reaction. Responder peripheral blood mononuclear cells (PBMC) were co-cultured with allogeneic T cell-depleted PBMC for 5 days, in the presence or absence of CTLA4Ig (1 or 100 µg/ml). Carboxyfluorescein succinimidyl ester dilution and percentages of CTLA4^+^ and PD1^+^ populations were determined after gating on CD4^+^. **(B)** Mean percentages of CD4^+^CTLA4^+^ and CD4^+^PD1^+^ T cells following allo-stimulation in the presence or absence of CTLA4Ig. Bars represent means + 1 SD (*n* = 4 independent experiments); *p < 0.05; only significant values are shown.

Next, we examined whether CD28 Co-SB could reduce CTLA4 and PD1 expression by allo-stimulated rhesus CD4^+^ T cells. As shown in Figure 4, in the absence of CTLA4Ig, both PD1 and CTLA4 expression was upregulated significantly by total (proliferating and non-proliferating) CD4^+^ T cells. In the presence of CTLA4Ig, reduced CD4^+^ T cell proliferation was associated with concentration-dependent reductions in the percentage of CTLA4^+^CD4^+^ T cells. In contrast, the incidence of PD1^+^CD4^+^ T cells not affected significantly.

### CD28 Co-SB Reduces Allo-Reactive CD4^+^CTLA4^hi^ T Cells *In Vitro*

**Figure 5:**
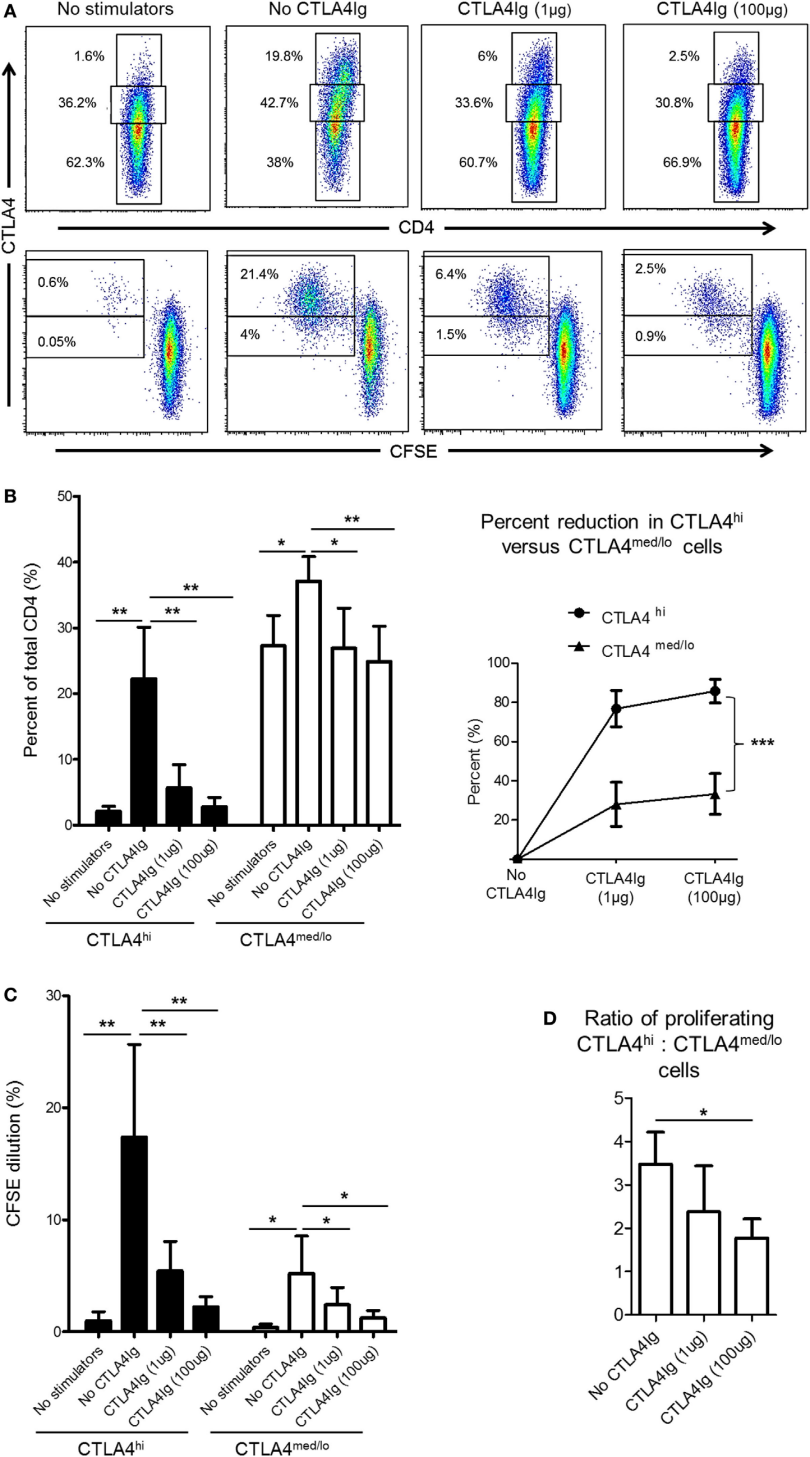
Cytotoxic T-lymphocyte-associated antigen (Ag) 4:Ig (CTLA4Ig) reduces CD4^+^CTLA4^hi^ more than CD4^+^CTLA4^med/lo^ T cells following allo-stimulation *in vitro*. **(A)** CTLA4Ig significantly reduces the incidence and proliferation of CD4^+^CTLA4^hi^ T cells following allo-stimulation *in vitro*. Carboxyfluorescein succinimidyl ester (CFSE)-labeled responder peripheral blood mononuclear cells (PBMC) were co-cultured with allogeneic, T cell-depleted PBMC for 5 days, in the absence or presence of CTLA4Ig (1 or 100 µg/ml). CFSE dilution of CTLA4^hi^ and CTLA4^med/lo^ CD4^+^ T cells and the percentages of total CTLA4^hi^ and CTLA4^med/lo^ CD4^+^ T cells were determined after gating on CD4^+^ cells. **(B)** Mean percentages of total CTLA4^hi^ and CTLA4^med/lo^ CD4^+^ T cells. **(D)** Percent reductions in CTLA4^hi^ and CTLA4^med/lo^ CD4^+^ T cells in the presence of CTLA4Ig are shown on the right. **(C)** Mean values of CFSE dilution of CTLA4^hi^ and CTLA4^med/lo^ CD4^+^ T cells (left). Ratios of CTLA4^hi^ to CTLA4^med/lo^ CD4^+^ T cell proliferation following allo-stimulation in the presence or absence of CTLA4Ig (right). Graphs represent data from five independent experiments; bars represent means + 1 SD. *p < 0.05; **p < 0.01; ***p < 0.001; only significant values are shown.

CD28 co-stimulation is essential for Treg differentiation, function, and homeostasis (41–43). In humans, Co-SB with CTLA4Ig decreases the incidence of circulating Treg (44, 45) and impairs Treg expansion (21). Thus, we questioned whether reduced CTLA4 expression by rhesus CD4^+^ T cells following allo-stimulation in the presence of CTLA4Ig was due mainly to enhanced suppression of the CTLA4^hi^ population. Following allo-stimulation in MLR, the frequencies of CD4^+^CTLA4^med/lo^ and CD4^+^CTLA4^hi^ among total CD4^+^ T cells were increased significantly (Figures 5A,B). However, these increases were more pronounced for CD4^+^CTLA4^hi^ (*p* < 0.01) than CD4^+^CTLA4^med/lo^ T cells (*p* < 0.05) (Figure 5B). The addition of CTLA4Ig during allo-stimulation significantly reduced the frequencies of both CD4^+^CTLA4^hi^ and CD4^+^CTLA4^med/lo^ populations in a concentration-dependent manner (Figure 5B; left). At CTLA4Ig 100 µg concentration, the frequencies of both populations were comparable with non-proliferating cells (Figure 5B; left). However, the percent reduction in CD4^+^CTLA4^hi^ T cells by CTLA4Ig was more marked than the percent reduction in CD4^+^CTLA4^med/lo^ T cells (*p* < 0.001) (Figure 5B; right).

While the reduction in CTLA4 expression mediated by CTLA4Ig could be attributed to reduced proliferation of both CD4^+^CTLA4^hi^ and CD4^+^CTLA4^med/lo^ T cell populations, a more pronounced reduction in CD4^+^CTLA4^hi^ (*p* < 0.01) than CD4^+^CTLA4^med/lo^ (*p* < 0.05) cell proliferation was observed (Figure 5C). To confirm this observation, we examined the ratio of proliferating allo-stimulated CD4^+^CTLA4^hi^ to CD4^+^CTLA4^med/lo^ T cells in the presence or absence of CTLA4Ig. CD4^+^CTLA4^hi^/CD4^+^CTLA4^med/lo^ T cell ratios were reduced significantly by CTLA4Ig in a concentration-dependent manner (Figure 5D). These observations indicate that CD28 Co-SB during allo-stimulation of normal rhesus CD4^+^ T cells *in vitro* may enhance the incidences of CD4^+^CTLA4^med/lo^ but not CD4^+^CTLA4^hi^ T cells.

### DCreg Infusion Maintains Donor-Specific CD4^+^CTLA4^hi^ T Cell Proliferation in CTLA4Ig-Treated Renal Allograft Recipients

**Figure 6:**
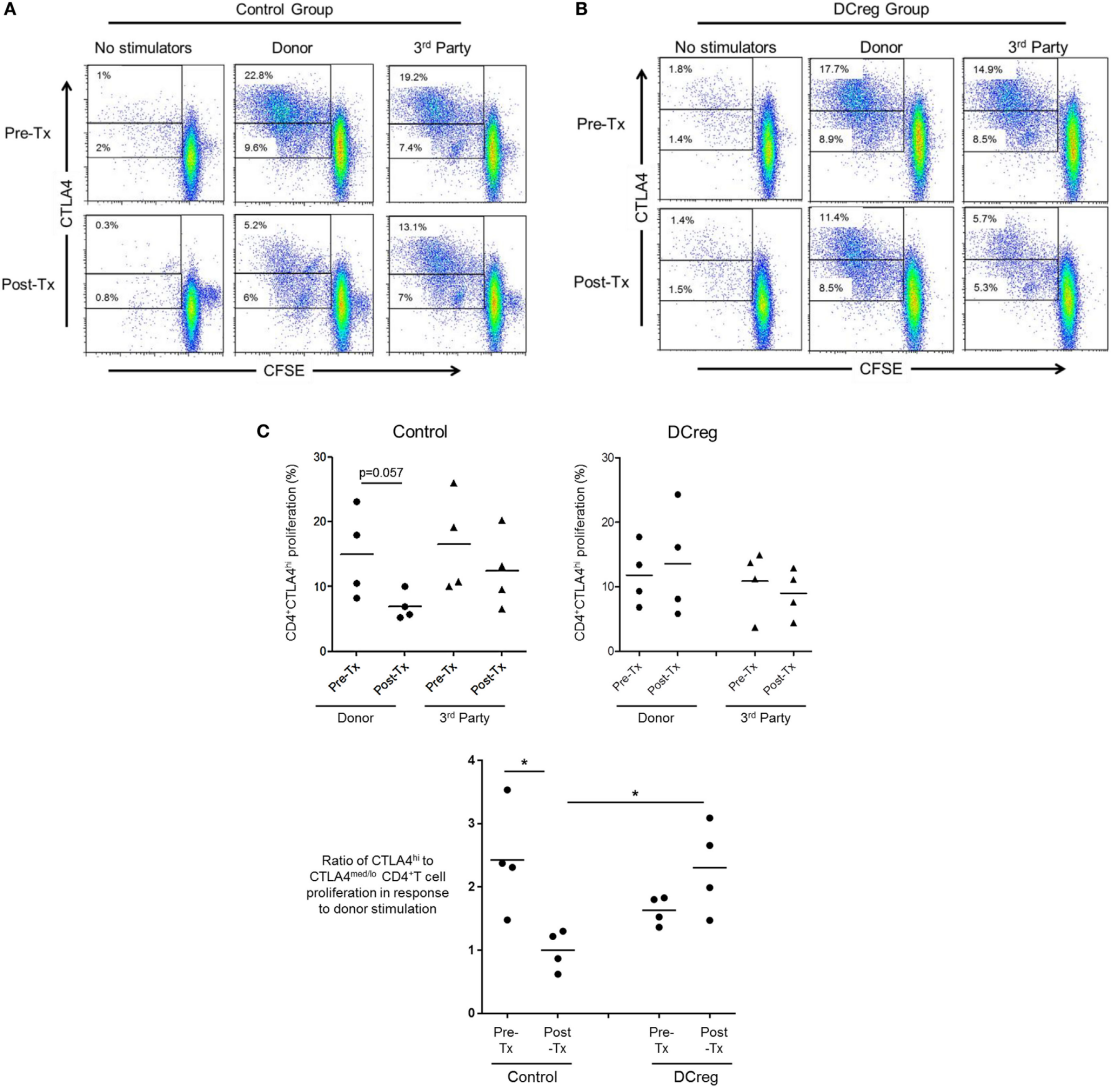
Regulatory dendritic cells (DCreg) infusion spares reduction of donor-specific CD4^+^CTLA4^hi^ T cell proliferation in cytotoxic T-lymphocyte-associated antigen (Ag) 4:Ig (CTLA4Ig)-treated renal allograft recipients. **(A)** In CTLA4Ig-treated renal allograft recipient with no DCreg infusion [control (CTRL) group], proliferation of CTLA4^hi^ and CTLA4^med/lo^ CD4^+^ T cells in response to donor or third party stimulation was measured before transplantation and at the time of euthanasia [post-operative day (POD) 28]. **(B)** Proliferation CTLA4^hi^ and CTLA4^med/lo^ CD4^+^ T cells in response to donor and third party stimulation in CTLA4Ig-treated renal allograft recipients with DCreg infusion (DCreg group) before transplantation and on POD 28 and POD 56. **(C)** Mean values of four graft recipients from the CTRL group (upper left) and four recipients from the DCreg group (upper right). In all recipients, ratios of percent proliferation of CTLA4^hi^ to CTLA4^med/lo^ CD4^+^ T cells in response to donor stimulation before and after transplantation are shown (below). *p < 0.05; only significant values are shown.

Our *in vitro* data suggest that CD28 Co-SB may be associated with a reduction in allo-reactive CD4^+^CTLA4^hi^ T cells following transplantation. In our earlier study (22), rhesus renal allograft recipients received CTLA4Ig together with rapamycin monotherapy, either without [control (CTRL) group] or in combination with pre-transplant donor-derived DCreg infusion (DCreg group). Since we observed upregulation of CTLA4 expression by host T cells in response to donor but not third party stimulation in recipients with DCreg infusion (22), we hypothesized that DCreg infusion before transplantation, would promote donor-reactive CD4^+^CTLA4^hi^ T cells after transplantation. We examined the proliferation of host CD4^+^CTLA4^hi^ and CD4^+^CTLA4^med/lo^ T cells in response to *ex vivo* donor or third party allo-Ag stimulation, before and 1 month after transplantation (Figure 6).

Before transplantation, proliferation of CD4^+^CTLA4^hi^ T cells in response to either donor or third party stimulation was similar in the CTRL and DCreg groups. In the CTRL group, the proliferation of CD4^+^CTLA4^hi^ T cells in response to donor stimulation was reduced markedly after transplantation (*p* = 0.057), but only modestly reduced in response to third party stimulation (Figure 6A). In contrast, in graft recipients given DCreg, proliferation of CD4^+^CTLA4^hi^ T cells in response to donor stimulation was not reduced after transplantation, whereas proliferation induced by third party cells was reduced modestly. Since there was no reduction in CD4^+^CTLA4^med/lo^ T cell proliferation in either group after transplantation compared with before transplantation (not shown), we evaluated the ratio of CD4^+^CTLA4^hi^ to CD4^+^CTLA4^med/lo^ proliferating T cells (as in Figure 5B) in response to donor stimulation in both groups (Figure 6C). With no DCreg infusion (CTRL), the proliferating CD4^+^CTLA4^hi^/CD4^+^CTLA4^med/lo^ T cell ratio in response to donor stimulation was reduced markedly after transplantation (*p* < 0.05). On the other hand, this ratio was slightly increased in graft recipients given DCreg infusion. Notably, in response to donor stimulation, the CD4^+^CTLA4^hi^/CD4^+^CTLA4^med/lo^ ratio was significantly higher in the DCreg-treated recipients than in the CTRL group (*p* < 0.05).

These observations indicate that, while CD28 Co-SB (with CTLA4Ig) can significantly reduce rhesus allo-reactive CD4^+^CTLA4^hi^ T cells both *in vitro* and in CTLA4Ig-treated renal allograft recipients, pre-transplant donor DCreg infusion prevents reduction of donor-specific CD4^+^CTLA4^hi^ T cells after transplantation.

## Discussion

We have reported previously (22) that a single systemic infusion of donor-derived DCreg 1 week before transplantation in combination with CD28 Co-SB (CTLA4Ig), results in prolonged renal allograft survival in a robust, MHC-mismatched pre-clinical NHP rhesus macaque model. Furthermore, graft recipients given DCreg showed selective attenuation of donor-reactive Tmem, associated with enhanced expression of the T cell co-inhibitory molecules CTLA4 and PD1 upon *ex vivo* stimulation of Tmem with donor but not third party cells.

Cytotoxic T-lymphocyte-associated Ag 4, a CD28 homolog, is upregulated by T cells after activation and negatively regulates immune responses (24, 27, 46). Rodent studies have validated the significance of CTLA4 expression for the promotion of allograft tolerance. Thus, blockade of the interaction between CTLA4 and B7 molecules accelerates graft rejection (28). Of particular relevance to our previous study in NHP (22), exposure of CTLA4-expressing CD4^+^ T cells to donor Ag is essential for the prevention of effector T cell responses and the promotion of transplant tolerance (47, 48). CTLA4 is expressed by natural and inducible Treg and contributes to their suppressive function (49, 50). Indeed, CTLA4 expression is critical for optimal Treg function and for the suppressive effect of these cells on T cell responses to allo-Ags (38, 39). While Foxp3 is known to upregulate CTLA4 expression (40), it has been argued that both Foxp3 and CTLA4 can independently promote immune tolerance (30).

CD28 co-stimulation is not only required for T cell activation and effector function, but it is also critical for Treg generation and for sustaining a balance between effector and Treg cells. CD28 co-stimulation and CTLA4 interaction with CD80 and CD86 are known to reduce CD4^+^ (51) and CD8^+^ (52) Th17 differentiation (53). CD28 Co-SB with CTLA4Ig (belatacept) was approved by the FDA in 2011 for kidney transplantation, but its use has been associated with higher rates of acute cellular rejection, despite superior renal graft function (16, 54). While allo-reactive Tmem are known to be CD28 Co-SB-resistant (18, 19) since they do not require co-stimulation (55, 56), some potentially unfavorable effects of CTLA4Ig on regulation of allo-reactive T cell responses are being recognized. Thus, recent reports indicate that CTLA4Ig can prevent Treg-dependent transplantation tolerance (20, 21). Moreover, in patients with rheumatoid arthritis (44) and following kidney transplantation (45), treatment with CTLA4-Ig has been shown to decrease the incidence of circulating Treg. These observations imply potential limitations of CTLA4Ig-based therapies for transplantation.

In our NHP renal allograft model (22), immunosuppression based on CD28 Co-SB using CTLA4Ig did not lead to increased Treg frequency, with or without DCreg infusion. However, we did observe increased Treg/Tmem ratios in the blood of graft recipients with DCreg infusion, in association with upregulation of CTLA4 and PD1 expression by Tmem following their stimulation with donor but not third party Ag. This suggests that, when Tmem encounter donor Ag following infiltration of the graft, they may upregulate PD1 and CTLA4, which in turn may control their activation/survival. Indeed, similar to graft-infiltrating CD8^+^ T cells, graft-infiltrating CD4^+^ T cells also upregulated CTLA4 and PD1 expression in DCreg-infused recipients (Figure 1).

In this study, we examined the relationship between CTLA4 and/or PD1 expression by allo-reactive T cells and Treg *in vitro* and observed that only CD4^+^ T cells with high CTLA4 expression exhibited a regulatory phenotype, i.e., CD25^hi^ Foxp3^hi^ CD127^lo^, while CTLA4^med/lo^ CD4^+^ T cells did not (Figure 4). On the other hand, there was no correlation between PD1 expression and Treg phenotype. Interestingly, while the frequencies of both CTLA4^hi^ and CTLA4^med/lo^ CD4^+^ T cells increased following allo-stimulation, CTLA4Ig reduced CTLA4^hi^ more than CTLA4^med/lo^ CD4^+^ T cells (Figure 5), resulting in the ratio of proliferating CD4^+^CTLA4^hi^ to CD4^+^CTLA4^med/lo^ T cells being reduced significantly in a CTLA4Ig concentration-dependent manner (Figure 5). This suggests that in normal rhesus, blocking CD28 co-stimulation during allo-stimulation reduces CD4^+^CTLA4^hi^ T cells in favor of CD4^+^CTLA4^med/lo^ T cells.

While in NHP allograft recipients, we found no increase in the frequency of circulating Treg, with or without DCreg infusion (22), there was a significant reduction in the incidence of circulating CD4^+^CTLA4^hi^ T cells in the control group following donor Ag stimulation post-transplant, while in recipients given DCreg infusion, the incidence of CD4^+^CTLA4^hi^ T cells was modestly elevated (Figure 6). Moreover, the ratio of CD4^+^CTLA4^hi^ to CD4^+^CTLA4^med/lo^ T cell proliferation in response to donor stimulation post-transplant was significantly higher in DCreg-treated than in control graft recipients. These observations suggest that an unfavorable influence of CTLA4Ig on allo-reactive Treg was averted in graft recipients given DCreg infusion. Indeed, combination of donor-derived DCreg with CTLA4Ig can result in long-term murine organ allograft survival, mediated at least in part, by CD4^+^ Treg (57).

Our observations indicate that high CTLA4 expression by donor-reactive T cells correlates with host immune regulation and is associated with better graft outcomes after transplantation. In a recent report (58), high levels of CTLA4 expression correlated with augmentation of CD4^+^ Th17 Tmem responses in renal allograft recipients given CD28 Co-SB immunosuppression. In our NHP study, we have confirmed the regulatory phenotype of CD4^+^CTLA4^hi^ T cells, compared with CD4^+^CTLA4^med/lo^ T cells. Although we did not assess Th17 expression in this study, donor-reactive CD4^+^CTLA4^med/lo^ T cells may correlate with Th17 CD4^+^ Tmem, particularly in the presence or absence of DCreg infusion.

Our observations provide further insight to the limitations of CD28 Co-SB in renal allo-transplantation. They are consistent with the view that, while CTLA4Ig efficiently prevents effector T cell responses to donor Ags, this may come at the expense of regulatory mechanisms that favor donor-specific Treg and attenuate donor-specific Tmem, and hence increased rates of acute cellular rejection. Our findings also suggest that DCreg infusion before renal transplantation is associated with preservation of donor-specific T cell regulation that may otherwise be compromised by CD28 Co-SB.

## Ethics Statement

All procedures were approved by the University of Pittsburgh Institutional Animal Care and Use Committee. Experiments were conducted according to the guidelines set forth in the National Institutes of Health Guide for the Care and Use of Laboratory Animals. Specific environment enrichment was provided.

## Author Contributions

ME participated in research design, writing of the paper, performance of the research, and data analysis. LL participated in performance of the research and data analysis. WS participated in performance of the research. AM participated in research design and data analysis. AT participated in research design, data analysis, and writing of the paper.

## Conflict of Interest Statement

The authors declare that the research was conducted in the absence of any commercial or financial relationships that could be construed as a potential conflict of interest.
